# Diagnostic Accuracy of Immunologic Biomarkers for Accurate Diagnosis of Bloodstream Infection in Patients with Malignancy: Procalcitonin in Comparison with C-Reactive Protein

**DOI:** 10.1155/2020/8362109

**Published:** 2020-10-31

**Authors:** Mansoureh Shokripour, Navid Omidifar, Kourosh Salami, Mohsen Moghadami, Babak Samizadeh

**Affiliations:** ^1^Department of Pathology, Shiraz University of Medical Sciences, Shiraz, Iran; ^2^Biotechnology Research Center, Shiraz University of Medical Sciences, Shiraz, Iran; ^3^Department of Internal Medicine, Shiraz University of Medical Sciences, Shiraz, Iran

## Abstract

**Purpose:**

To calculate the diagnostic value of C-reactive protein (CRP) and serum procalcitonin (PCT) levels for the pathologic presence of microbes in the bloodstream of patients with malignancy, in comparison with blood culture. *Methodology*. Blood culture (by reference method) and assay results of PCT and CRP of febrile patients, with clinical suspicion to blood infections, were collected. Statistical aspects of PCT  and CRP tests were evaluated.

**Results:**

Data from 255 cases were gathered. The area under the curve for differentiating bacteremia from nonbacteremia for PCT (0.741) was superior to that of CRP (0.612). Amongst the different cutoffs of PCT and CRP, the cutoff of ≥1.17 ng/ml and >47 mg/l had the sensitivity of 75 and 58.3%, the best NPV of 91.5% and 81.3%, and the best specificity of 79.9% and 72.8%, respectively. *Discussion*. Despite statistically nonsignificant results, PCT seems to be a superior indicator to CRP for rejecting the presence of microorganism in bloodstream. For PCT, the cutoff value of 1.17 ng/ml (bacteremia from nonbacteremia) had the highest NPV value of 91.5% in malignant patients, suspicion of sepsis.

## 1. Introduction

Infections are an important cause of morbidity and mortality amongst oncology patients, and neutropenia is recognized as the most important risk factor, which its severity and duration are associated with a higher rate of infection [1]. The classic symptoms of infection are fever and leukocytosis. Fever with a temperature greater than 38.3°C sublingually is alarming in cancer patients. Temperature greater than 38°C for more than an hour in a patient whose absolute neutrophil count (ANC) of peripheral blood is less than 500 cells per cubic millimeters or below is called neutropenia with fever. In these patients, since the immune system is suppressed by extensive chemical therapies, fever can be considered as the only positive finding as a probable infection that should be further investigated [2]. Fever is a specific, but not a sensitive marker, and is influenced by noninfectious factors such as antipyretics. However, it is a criterion for sepsis [3, 4]. Also, leukocytosis has limitations in the diagnosis of infection and sepsis [5].

CRP is a well-known marker in the field of sepsis, and its association with the treatment of urosepsis was detected [6, 7]. Use of this marker to detect infectious diseases amongst children and adults is proven [8]. CRP measurement is an inexpensive test available in almost all healthcare centers, and some researchers prefer it over other markers [9].

Procalcitonin (PCT) was first introduced in 1993 by Assicot et al. as a sepsis-induced protein, found in the plasma of patients with infection and sepsis. Early studies showed that this marker is mainly elevated in severe systemic inflammation by bacterial agents and is not increased in other types of inflammation, such as viral and autoimmune. Plasma values are very low in normal cases and are about 10 to 50 pg/ml [10, 11]. It can be used for the early detection of infections and also to monitor the response to a treatment [12]. Finally, gold standard for the identification of infections and bacteremia is microbial cultures, which also has some problems, for example, distinguishing between true bacteremia and contamination, polymicrobial cultures, and the timing of this method [13].

Selecting patients' with true bacteremia to initiate appropriate antibiotic therapy is important in controlling the mortality, especially in the oncology ward. Hence, we aimed to investigate the value of CRP and PCT to determine the value of each marker for early detection of true bacteremia for better management of these patients.

## 2. Methods and Materials

This study was performed on patients with clinical suspicion of infection in an oncology center in the city of Shiraz, Iran, in 2017-18. All of the patients with malignancy that had fever with a body core temperature of more than 37 degree were included in the study. If a patient was unwilling to cooperate or sample was unavailable, they were excluded from the study, and finally, 255 patients were included. Peripheral blood samples were taken before starting antibiotic treatment. One of the samples was sent for microbiological examination in BACTEC glass and the other in clot tube for evaluation of CRP and PCT markers. Another blood culture sample was taken from another site simultaneously. After taking cultures, empirical antibiotic therapy was started. Type of antibiotics was changed according to the results of antibiotic susceptibility testing. We did not change the antibiotics according the results of PCT/CRP. Antibiotic therapy was stopped 14 days after the first negative culture for Gram-positive bacteria and fungi. It was 21 days for Gram-negative bacteria. For a patient with negative culture, antibiotics were stopped 3 days after lowering the fever and stabilization of patient vital signs.

Demographic information, white blood cell count, and neutrophil count of patients were also recorded.

Peripheral blood BACTEC samples were analyzed by the BACTEC 9120 system. Positive samples were inoculated in 5% sheep blood agar, EMB agar, and chocolate agar and incubated for one day at 35–37°C. In case of microorganism growth, staining, colony morphology, biochemical tests, and automatic identification systems (API, bioMérieux, France) were used to identify the microorganism.

BACTEC samples were kept in the apparatus for up to 7 days if they did not grow. At the end of the seventh day, we considered microorganism growth as negative or nonbacteremia for cultures without organism growth (group I) and cultures with organism growth as bacteremia. In this group, bacteremia was divided into two groups: true bacteremia (group II) and contamination (group III). Identification of normal skin flora microorganisms, such as Staph coagulase negative, Corynebacterium, and *Streptococcus viridans*, in only one blood culture glass was considered as contamination. Coagulase-negative Staphylococci were considered to be true bacteremia, only when they are isolated in two blood cultures in two different times and with similar antibiotic susceptibility. Finally, we considered isolation of other microorganisms as true bacteremia. CRP levels were measured, using Bionik kit and BT 3000 autoanalyzer (based on latex immunoturbidometry). The PCT was measured on peripheral blood serum, using VIDAS product kit. Analytical sensitivity for PCT and CRP was 0.05 ng/ml and 0.05 mg/l, respectively.

Data were analyzed using SPSS version 9.1 and MedCalc version 8 software. One-way ANOVA statistical method was used to compare the mean of variables in different groups and the ROC curve to determine the value of PCT and CRP markers as well as sensitivity and specificity of different cutoffs. *P* values less than 0.05 were considered to be statistically significant. The local ethics committee of Shiraz University of Medical Sciences approved this study.

## 3. Results

Of the 255 investigated patients, 135 were male (52.9%) and 120 were female (47.1%). From the 255 patients, 234 were children (91.8%) and the rest were adults (8.2%). The mean age of the children was 7.3 ± 5.5 years, and the mean age of the adults was 36 ± 1.6 years.

In our study, we focused on all of the patients with malignancy and fever without any concentration on special type of malignancy. Most of the pediatric patients (164) had hematologic malignancy (70.08%) and also the same was true about the adult patients, hematologic malignancies (15, 71.42%). Detailed distribution of recruited patients are as follows: acute lymphoblastic leukemia (ALL), 97 (38.0%); acute myelogenous leukemia (AML), 62 (24.3%); non Hodgkin lymphoma (NHL), 17 (6.7%); Hodgkin lymphoma (HL), 1 (0.4%); neuroblastoma, 9 (3.5%); Wilms' tumor, 7 (2.7%); hepatoblastoma, 9 (2.7%); germ cell tumor, 8 (3.5%); PNET/Ewing's sarcoma, 20 (7.8%); rhabdomyosarcoma, 5 (2.0%); brain tumor, 10 (3.9%); lung adenocarcinoma, 1 (0.4%); colorectal cancer, 3 (1.2%); multiple myeloma (MM), 2 (0.8%); osteosarcoma, 3 (1.2%); and pleuropulmonary blastoma, 1 (0.4%).

From our patients, 88 of them (34.51%) had neutropenia with less than 500 neutrophiles per microliter by definition. It is named febrile neutropenia.

Blood culture was considered as the gold standard for the diagnosis of bacteremia. The most abundant microorganism species in the bacteremia group were as follows: *E. coli*, *coagulase-negative Staphylococcus*, and *non-albicans Candida*, with three cases of each, and the rest one case *Enterococcus*, *Group A Streptococci*, *group D hemolytic Streptococcus*, *Salmonella*, *Pseudomonas*, and *Candida albicans*. In the contamination group, in three patients, *Streptococcus viridans* organism and in one patient, *Micrococcus* organism were detected. Based on the criteria and clinic of these patients, they were grouped as contaminated.

The lowest and highest and mean of WBC levels in the bacterial and nonbacterial groups and the contamination are shown in Table 1. There was no significant difference between groups in WBC values (*P* value >0.05).

According to clinical conditions, there was a statistical significant difference in PCT concentration between the bacteremia group and nonbacteremia as well as the contamination group. Also, there was a statistical significant difference in the PCT level between Gram-negative group and Gram-positive as well as fungal group. There was no statistical difference in the CRP level between these abovementioned groups (Table 2).

**Figure 1:**
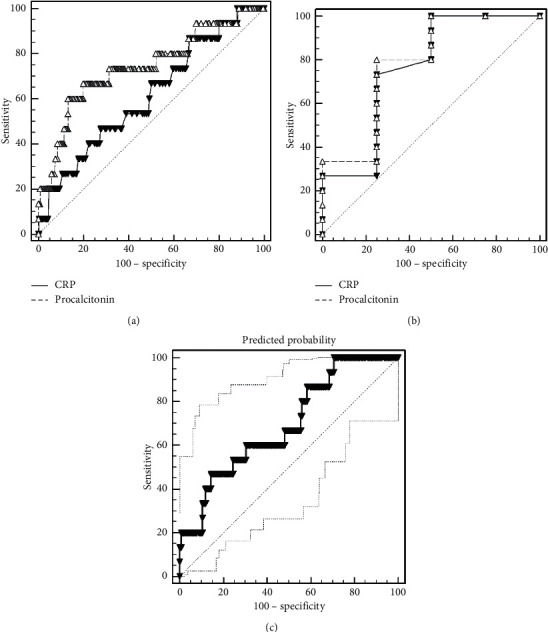
ROC curve of PCT and CRP in diagnosing bloodstream infections in oncology patients (a) between bacteremia and nonbacteremia groups, (b) between bacteremia and contamination groups, and (c) combined PCT and CRP between bacteremia and nonbacteremia groups.

The ROC curve was used to compare the value of these two markers to distinguish bacteremia from nonbacteremia and also bacteremia from contamination (Table 3). Only PCT and combination of PCT and CRP were statistically significant in distinguishing bacteremia from nonbacteremia patients. However, significant difference was not seen between the AUC values of PCT and those of CRP (*P* value: 0.183).  Figure 1 displays the ROC curves of the PCT and CRP for discriminating bacteremia from other groups. We also compared the sensitivity of both markers in these two groups at fixed specificities of 50%, 75%, and 98% (Table 4). As shown, the sensitivities of PCT at different specificities are higher than CRP.

Using ROC curve results, the new CRP cutoff and PCT cutoff to distinguish bacteremia from nonbacteremia was 47 mg/dl and 1.17 ng/ml, respectively, with sensitivity, specificity, PPV, and NPV that are shown in Table 5.

## 4. Discussion

In infections, CRP and PCT are used as diagnostic markers. Due to the importance of rapid diagnosis of sepsis in immunosuppressed oncology patients, it is imperative to find a marker that can quickly and accurately predict the probability of sepsis and bacteremia. In this study, we evaluated the value of CRP marker and PCT in differentiating bacteremia from nonbacteremia as well as differentiating bacteremia from contamination in peripheral blood cultures. The results showed that the mean value of the PCT level was significantly higher in the bacteremia than the nonbacteremia group, and this value was also significantly higher in the bacteremia than the contamination group. However, there was no significant difference in CRP values between these bacterial and nonbacterial as well as contamination groups. Also, in comparison between bacteremia and nonbacteremia, the AUC of PCT was higher than CRP, and the *P* value of PCT as a single test was statistically significant, but it is not for CRP. In fixed specificities, the sensitivities of PCT were higher than CRP in bacteremia versus nonbacteremia groups. All of these findings are in favor of a greater value of PCT to distinguish bacteremia from nonbacteremia, which might be due to increased production of CRP in cancer, especially hematologic malignancies [14]. Similar to our findings, several other studies also reported the same thing on oncology patients [14–20]. In a number of studies in contrast to ours and previous studies, it is stated that there is no statistical difference between PCT values in comparison with CRP [21–24]. It may be due to the use of old assay methods with poor analytical sensitivities.

In our study, the difference in PCT averages in Gram-negative bacteremia in comparison with Gram-positive as well as fungal groups was statistically significant, which was confirmed by most of the previous studies [14–20]. In contrast, in few studies, this difference of PCT in Gram-negative bacteremia was not statistically significant [23, 24]. Between these groups, CRP was not statistically significant.

According to ROC analysis for PCT, at the optimal cutoff value of 1.17 ng/ml (bacteremia from nonbacteremia) in comparison with a standard cutoff value of 0.5, the NPV value was 97.46% with a higher specificity of 80.08% in malignant patients, suspicious of sepsis. With this higher cutoff value, with more certainty, we can exclude blood infections in oncology patients.

In this study, we found few cases with a significant increase in PCT levels, which had negative peripheral blood cultures. In clinical studies, other causes such as urosepsis and involvement with invading fungi such as *Aspergillus* might attribute to elevated levels of this marker in the absence of bacteremia. However, given the context of immunosuppression amongst our patients and the possibility of respiratory and urinary infection as well as the effect of chemotherapy drugs, these two inflammatory markers can be elevated without the presence of clear bacteremia. In addition, transient bacteremia and inappropriate timing might be the other causes of these inconsistencies between high levels of CRP and PCT with peripheral blood cultures.

One significant limitation of our study was very low number of contamination group cases which our suggestion is a study with more number of contaminated cases before and after good training of personnel.

In conclusion, according to recent study, there was a significant difference between the mean of PCT in the bacteremia and nonbacteremia group, and lack of this difference for CRP showed that PCT was better in differentiating bacteremia from nonbacteremia and also in Gram-negative cases versus other positive cultures in patients with malignancy.

## Figures and Tables

**Table 1:** Lowest and highest WBC averages across groups.

WBC/mm^3^	Lowest	Highest	Mean
Nonbacteremia	30	77700	6059
Bacteremia	60	25000	5930
Contamination	2700	8500	6500
Gram-positive	200	25000	9800
Gram-negative	60	13900	3512
Fungal	140	7700	3150

**Table 2:** The lowest and highest and mean of CRP and PCT concentrations.

Result	Count	CRP mean (lowest-highest)	ANOVA (*P* < 0.274)	PCT mean (lowest-highest)	
Nonbacteremia (group I)	236	62.27 (2–211)		1.47 (0.27–47)	

Bacteremia (group II)	15	76.6 (18–141)	>0.05	13.93 (0.12–100)	
Gram-positive (G+)	6	84.77 (29–121)	>0.05	1.7 (0.22–4.32)	
Gram-negative (G−)	5	62.4 (18–108)	>0.05	28.7 (0.2–100)	
Fungal (F)	4	82.25 (56–141)	>0.05	13.7 (0.12–52.09)	

Contamination (group III)	4	42.50 (6–99)	>0.05	1.10 (0.1–3.9)	

*P* value for PCT between groups					

I vs. II	II vs. III	I vs. III	G− vs. G+	G− vs. F	G+ vs. F

0.000	0.013	0.995	0.000	0.028	0.096

**Table 3:** PCT and CRP area under ROC curve value between bacteremia and nonbacteremia groups.

Groups	Parameter	AUC	95% CI	*P* value
*Bacteremia vs. nonbacteremia*	CRP	0.612	0.549–0.673	0.1463
	PCT	0.741	0.682–0.794	0.0015
	Combined PCT and CRP	0.693	0.632 to 0.749	0.0058

*Bacteremia vs. contamination*	CRP	0.758	0.921–0.511	0.8167
	PCT	0.783	0.538–0.935	0.8625

**Table 4:** Sensitivity and cutoff level of PCT and CRP at fixed specificities between bacteremia and nonbacteremia groups.

Marker		Specificity		
		50	75	98
*PCT*	Cutoff (ng/ml)	0.36	0.899	13
	Sensitivity %	73.33	66.67	20

*CRP*	Cutoff (ng/ml)	61	84	126
	Sensitivity %	66.67	40	6.67

**Table 5:** Sensitivity and specificity and PPV and NPV of CRP and PCT markers to distinguish bacteremia from nonbacteremia at optimal cutoff in comparison with standard cutoff based on ROC curve results.

Marker		Cutoff	Sensitivity (95% CI) in %	Specificity (95% CI) in %	PPV in %	NPV in %
*PCT*	Standard cutoff (ng/ml)	0.5	73.33 (44.9–92.2)	61.86 (55.3–68.1)	10.76	97.37
	Optimal cutoff (ng/ml)	1.17	66.67 (38.4–88.2)	80.08 (74.4–85)	17.34	97.46

*CRP*	Standard cutoff (mg/l)	10	100 (78.2–100)	7.63 (4.6–11.8)	6.36	100
	Optimal cutoff (mg/l)	47	86.67 (59.5–98.3)	33.05 (27.1–39.4)	7.5	97.53

## Data Availability

The data supporting the findings of this study are available within the article.
